# HIV Glycoprotein Gp120 Impairs Fast Axonal Transport by Activating Tak1 Signaling Pathways

**DOI:** 10.1177/1759091416679073

**Published:** 2016-11-20

**Authors:** Sarah H. Berth, Nichole Mesnard-Hoaglin, Bin Wang, Hajwa Kim, Yuyu Song, Maria Sapar, Gerardo Morfini, Scott T. Brady

**Affiliations:** 1 Department of Anatomy and Cell Biology, University of Illinois at Chicago, IL, USA; 2 Marine Biological Laboratory, Woods Hole, MA, USA; 3 Center for Clinical and Translational Sciences, University of Illinois at Chicago, IL, USA; 4 Department of Systems Biology and Laboratory of Systems Pharmacology, Harvard Medical School, Boston, MA USA; 5 Department of Biological Sciences, Howard Hughes Medical Institute, Hunter College, New York, NY, USA

**Keywords:** axonal transport, distal sensory polyneuropathy, gp120, HIV, mitogen-activated protein kinase, kinesin

## Abstract

Sensory neuropathies are the most common neurological complication of HIV. Of these, distal sensory polyneuropathy (DSP) is directly caused by HIV infection and characterized by length-dependent axonal degeneration of dorsal root ganglion (DRG) neurons. Mechanisms for axonal degeneration in DSP remain unclear, but recent experiments revealed that the HIV glycoprotein gp120 is internalized and localized within axons of DRG neurons. Based on these findings, we investigated whether intra-axonal gp120 might impair fast axonal transport (FAT), a cellular process critical for appropriate maintenance of the axonal compartment. Significantly, we found that gp120 severely impaired both anterograde and retrograde FAT. Providing a mechanistic basis for these effects, pharmacological experiments revealed an involvement of various phosphotransferases in this toxic effect, including members of mitogen-activated protein kinase pathways (Tak-1, p38, and c-Jun N-terminal Kinase (JNK)), inhibitor of kappa-B-kinase 2 (IKK2), and PP1. Biochemical experiments and axonal outgrowth assays in cell lines and primary cultures extended these findings. Impairments in neurite outgrowth in DRG neurons by gp120 were rescued using a Tak-1 inhibitor, implicating a Tak-1 mitogen-activated protein kinase pathway in gp120 neurotoxicity. Taken together, these observations indicate that kinase-based impairments in FAT represent a novel mechanism underlying gp120 neurotoxicity consistent with the dying-back degeneration seen in DSP. Targeting gp120-based impairments in FAT with specific kinase inhibitors might provide a novel therapeutic strategy to prevent axonal degeneration in DSP.

## Introduction

Distal sensory polyneuropathy (DSP) is a frequent complication of human immunodeficiency virus (HIV) infection that has persisted despite tight viral control by combination antiretroviral therapy (Kranick and Nath, 2012). A major pathological characteristic of DSP is progressive, dying-back degeneration of dorsal root ganglion (DRG) axons (Keswani et al., 2002). DSP patients suffer from excruciating pain (Dorsey and Morton, 2006); yet, there is a dearth of effective treatments (Phillips et al., 2010). A significant body of research has documented the neurotoxicity of the HIV glycoprotein gp120 (Brenneman et al., 1988; Toggas et al., 1994; Lannuzel et al., 1995; Meucci and Miller, 1996; Hesselgesser et al., 1998; Meucci et al., 1998; Singh et al., 2005; Kaul et al., 2007; Bachis et al., 2009), which is overproduced and shed from the viral capsid (Schneider et al., 1986). Further, evidence has accumulated for direct toxicity by gp120 on DRG neurons (Hesselgesser et al., 1998; Bodner et al., 2004; Wilkerson et al., 2012), suggesting that gp120 might play a causal role in DSP.

While gp120 has been shown to cause axonal degeneration (Melli et al., 2006; Robinson et al., 2007) and apoptosis (Hesselgesser et al., 1998; Bachis et al., 2003; Bodner et al., 2004; Bachis et al., 2006), the underlying mechanisms upstream of these events remain to be elucidated. Interestingly, recent experimental evidence showing internalization and intra-axonal location of gp120 in DRG neurons (Berth et al., 2015) suggests the possibility of neurotoxicity by intra-axonal gp120.

The extreme polarization of DRG neurons with long axons places remarkable transport demands on these cells, since virtually all proteins must be transported from their site of synthesis in the neuronal cell bodies. This daunting task depends upon fast axonal transport (FAT), a cellular process critical for proper functioning and maintenance of axons and synapses (Morfini, 2012). Significantly, genetic and experimental evidence linked impairments in FAT to axonal degeneration in various diseases featuring dying-back degeneration of neurons (Morfini, You, et al., 2009). The slowly progressive and subclinical manifestation of DSP (Keswani et al., 2002) follows an established pattern in dying-back neuropathies linked by dysregulation of FAT, termed dysferopathies (Morfini, You, et al., 2009). In a DSP model using simian immunodeficiency virus-infected macaques, distal axons of peripheral nerves showed an accumulation of damaged mitochondria (Lehmann et al., 2011), suggestive of alterations in FAT. Additionally, gp120 was found to activate the stress-activated protein kinases, p38 mitogen-activated protein kinase (MAPK), and c-Jun N-terminal Kinase (JNK) in rodent models of DSP (Milligan et al., 2001; Bodner et al., 2004; Wilkerson et al., 2012), and these kinases are known to regulate FAT by phosphorylating molecular motors (Morfini et al., 2006; Morfini et al., 2013). Together, these precedents raised the possibility that gp120 might alter FAT by promoting abnormal activation of protein kinases. However, effects of gp120 on FAT have not been examined.

The aim of this study was to define the effect of intra-axonal gp120 on FAT. Studies using the isolated squid axoplasm preparation indicated that gp120 profoundly impaired both anterograde and retrograde FAT through abnormal activation of regulatory kinases for FAT. With the finding that both p38 MAPK and JNK mediated inhibition of FAT, the upstream activator of p38 MAPK and JNK was narrowed down to the MAP3K Tak-1. Interestingly, gp120 was also found to activate inhibitor of kappa B kinase (IKK)-2 downstream of Tak-1, which represents a novel regulatory pathway for FAT. Extending these findings to mammalian sensory neurons, axonal growth assays confirmed a critical role of Tak1 in the axonal pathology associated with gp120 treatment. Collectively, these studies demonstrate a novel toxic mechanism for gp120 that is consistent with the dying-back pattern of neuronal degeneration in DSP.

## Materials and Methods

### Antibodies and Reagents

The following primary antibodies were used: anti-KHC (H2 clone; [DeBoer et al., 2008]), anti-p38 MAPK (Cell Signaling #9212), anti-phospho-p38 MAPK (Cell Signaling #9215), anti-dynein intermediate chain 74-1 clone antibody (Santa Cruz #sc-13524), and anti-tubulin DM1A clone antibody (Sigma–Aldrich #T9026). The following secondary antibodies were used: Jackson 111-035-062 HRP-conjugated goat anti-mouse IgG, Jackson 111-035-144 HRP-conjugated goat anti-rabbit IgG, and Amersham PA45010 Cy5-tagged goat anti-mouse IgG. AMD3100 (AMD) and gp120 BaL were obtained from the National Institutes of Health AIDS Research and Reference Reagent Program. Recombinant gp120 IIIB was purchased from Immunodiagnostics (#1001). SB203580 (#559389), okadaic acid (#495620), SP600125 (#420119), I-2 (#14-162), (5Z)-7-oxozeaenol (#499610), and IKK Inhibitor XII (#401491) were obtained from Calbiochem. MW01-2-069A-SRM is a small molecule drug developed at Northwestern University and described in (Munoz et al., 2007). Recombinant IKK-2 was obtained from Signal Chem. The DVD peptide was synthesized at the UIC Biologic Resources Laboratory facility. ING-135 was a gift from Allen Kozikowski (UIC), and CEP-11004 was a gift from Cephalon.

### F11 Cell Culture

F11 cells (a generous gift from Dr. Richard Miller of Northwestern University) were grown in high glucose DMEM media (Invitrogen), supplemented with 10% fetal bovine serum, 10% glutamax, and 10,000 U/ml penicillin-streptomycin; 100 mm-petri dishes were coated with 0.1 µg/ml poly-L-lysine (Sigma) and rinsed four times with autoclaved, deionized water for half an hour each; and 150,000 cells were added to each petri dish. Cells were maintained at 37 ℃ in 5% CO_2_ and 95% O_2_. To differentiate the F11 cells, 24 hr after plating the amount of fetal bovine serum was reduced to 5%, and then 24 hr after that the cells were treated with 0.5 mM dibutryl-cAMP (Sigma) in media with 0.5% fetal bovine serum for 4 days.

### Cell Lysates

After 4 days of 0.5 mM dibutryl-cAMP (Sigma) treatment, F11 cells were treated with 5 nM gp120 (Immunodiagnostics) for 5, 15, or 30 min. An experimental group of cells were first treated with 2 µM AMD3100 for 1 hr at 37℃ prior to treatment with 5 nM gp120 for 5, 15, or 30 min. Treatment with phosphate-buffered saline (PBS), the gp120 diluent, was used as a negative control. After treatment, cells were scraped and collected in 700 μL lysis buffer (1% sodium dodecyl sulfate in PBS, pH 7.4) and sonicated for two 3-sec bursts each. Cells were spun at 14,000 rpm at 4℃ in a Beckman tabletop centrifuge. Protein concentration of clarified lysates was determined using a bicinchoninic acid assay kit (Pierce). Sample buffer was added to the clarified supernatants, and the samples were placed in the −80 ℃ freezer prior to analysis via sodium dodecyl sulfate polyacrylamide gel electrophoresis.

### Immunoblots

Protein samples were separated using 4%–12% bis/tris MOPS gradient gel (Invitrogen) and transferred to Immobilon-P transfer membranes (polyvinylidene fluoride, Millipore) at 0.4 Amps for 2 hr in 1X Tobin buffer. Membranes were blocked at room temperature for 60 min with 5% (w/v) nonfat dried milk in PBS or tris-buffered saline (TBS) with 0.1% Tween-20 for phospho-p38 MAPK antibody. Sodium orthovanadate (1 mM) and sodium fluoride (10 mM) were added to TBS in all incubation steps involving the use of phosphoantibodies. Primary antibodies in 1% bovine serum albumin (BSA) or 5% BSA (phospho-p38 MAPK) were added overnight at 4 ℃ with gentle rocking. Primary antibodies were washed 3 × 10 min with PBST (0.25% Tween-20) or TBST (0.1% Tween-20), and secondary antibodies were added for 1 hr at 4℃ with gentle rocking. Membranes were again washed 3 × 10 min and visualized with ECL (Amersham) and exposed on film (Kodak) for HRP secondary. Immunoreactive band intensities within linear range were quantitated and analyzed using Quantity One software (Bio-Rad Laboratories). The student's *t* test was used for statistical analysis. Quantitative data were expressed as mean ± *SEM*, and significance was determined at *p* < .05.

### Vesicle Motility Assays in Isolated Squid Axoplasm

Axoplasms were extruded from giant axons of the squid *Loligo pealii* (Marine Biological Laboratory) as described (Song et al., 2016). Gp120 IIIB or gp120 BaL was diluted into X/2 buffer (175 mM potassium aspartate, 65 mM taurine, 35 mM betaine, 25 mM glycine, 10 mM HEPES, 6.5 mM MgCl_2_, 5 mM EGTA, 1.5 mM CaCl_2_, 0.5 mM glucose, pH 7.2) supplemented with 5 mM ATP and 20 µl added to perfusion chambers. Preparations were analyzed on a Zeiss Axiomat with a 100×, 1.3 n.a. objective, and differential interference contrast optics. Hamamatsu Argus 20 and Model 2400 CCD camera were used for image processing and analysis. Organelle velocities were measured with a Photonics Microscopy C2117 video manipulator (Hamamatsu). All experiments were repeated at least three times. For statistical analysis in Figures 2(e), 3(d), 4(e), and 5(f), data from 30- to 50-min time points were pooled. To compare gp120 treatment to heat-inactivated gp120, Student's two-tailed *t* test was used. Analysis of variance (ANOVA) was utilized to compare the effects of different inhibitors to both buffer control and to 10 nM gp120 treatment. Quantitative data were expressed as mean ± *SEM*, and significance was determined at *p* < .05.

**Figure 1.**
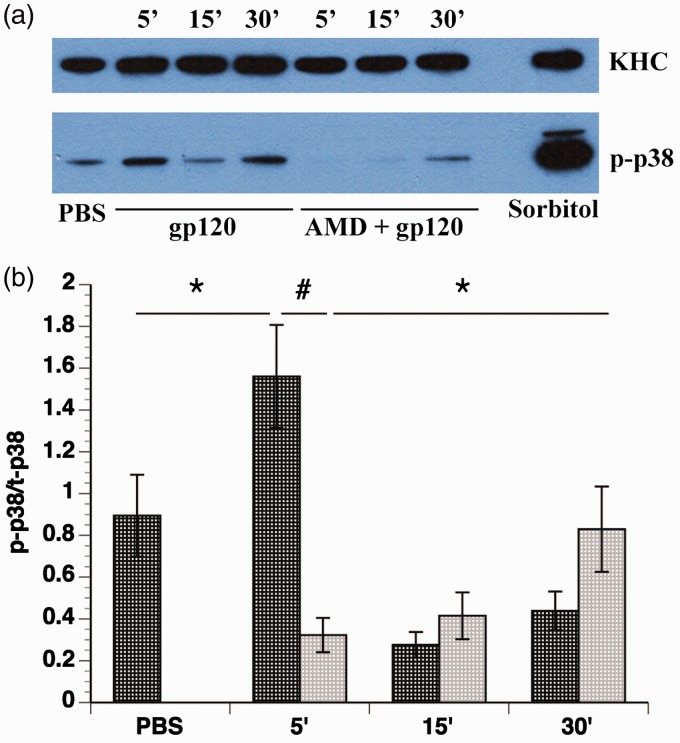
Activation of p38 MAPK by gp120 treatment. Differentiated F11 cells were treated with 5 nM gp120 for 5, 15, and 30 min. The same volume of PBS, the diluent, was added as a control, and 0.5 M sorbitol treatment (5 min) was used as a positive control for p38 MAPK activation. To examine the contribution of CXCR4 activation on p38 MAPK activation, differentiated F11 cells were incubated with 2 µM AMD3100 for 1 hr prior to gp120 treatment. (a) A representative Western blot shows p38 MAPK activation, as shown by an antibody recognizing phosphorylated (active) p38 MAPK (p-p38). The H2 antibody for kinesin heavy chain (KHC) was used as a loading control. (b) Quantitation of p38 MAPK activation. Dark gray bars represent no AMD3100 pretreatment, and light gray bars represent AMD3100 pretreatment. The amount of phosphorylated p38 MAPK was significantly increased after 5 min of gp120 treatment compared with control (*p* < .05). This transient activation was abolished by pretreatment with AMD3100 (*p* < .0001) and was therefore dependent upon CXCR4 activation. However, AMD3100 treatment unmasked a CXCR4-independent activation of p38 MAPK after 30 min of treatment (*p* < .05). Data represent the mean *± SEM* of the ratio of phospho-p38 MAPK to total p38 MAPK from 16 separate experiments. *p < .05. ^#^*p* < .0001.

**Figure 2.**
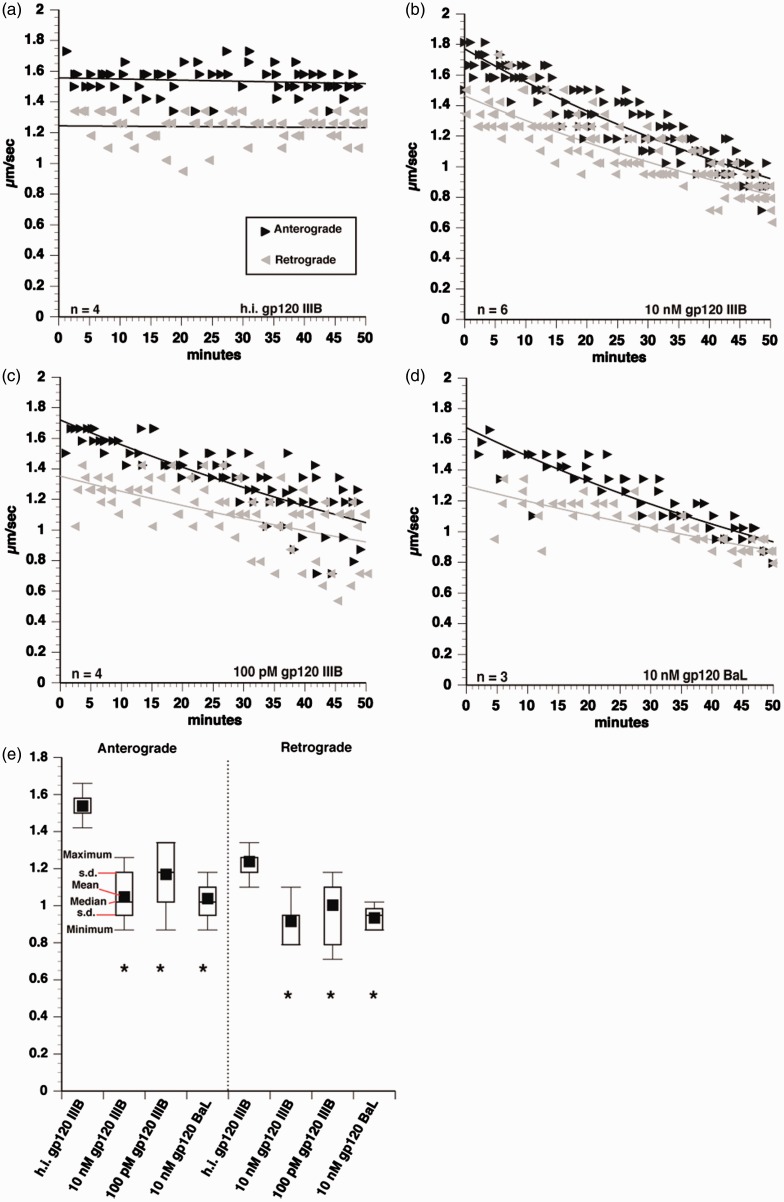
Inhibition of anterograde and retrograde FAT by gp120. Vesicle motility assays in squid axoplasm. Individual velocity (µm/sec) rate measurements (arrowheads) are plotted as a function of time (minutes). Black arrowheads and lines represent anterograde, conventional kinesin-dependent FAT rates. Gray arrowheads and lines represent retrograde, CDyn-dependent FAT rates. Perfusion of 10 nM heat-inactivated gp120 IIIB had no effect on FAT (a), but 10 nM gp120 IIIB profoundly impaired FAT in both anterograde and retrograde directions (b). The effect of 100 pM gp120 IIIB FAT was comparable to that of 10 nM (c), demonstrating that a physiologically relevant concentration inhibits FAT. Additionally, perfusion of a CCR5-preferring strain, BaL, showed the same impairment of FAT (d), indicating that the effect of FAT inhibition by gp120 is conserved across strains. Box plots were generated using pooled values from 30- to 50 min time points for statistical comparisons of each treatment (e). A student's two-tailed *t* test performed using pooled values from 30- to 50 min time points demonstrated that 10 nM gp120 IIIB, 100 pM gp120 IIIB, and 10 nM gp120 BaL all significantly inhibit both anterograde and retrograde FAT, compared with heat-inactivated gp120. *p < .001.

**Figure 3.**
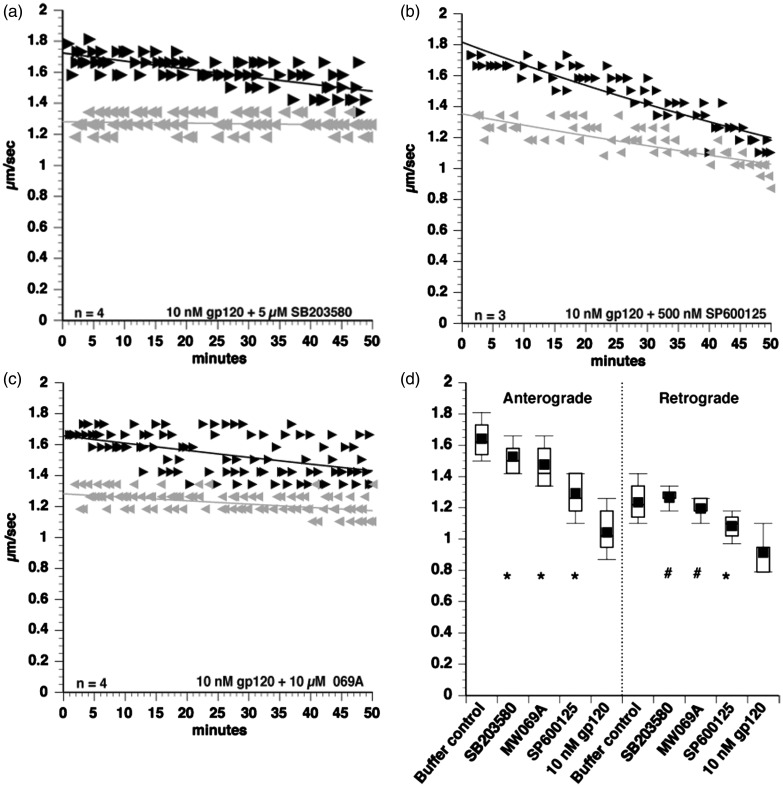
Gp120 activates p38 MAPK and JNK to impair FAT. To identify kinases mediating the toxic effect of gp120 IIIB on FAT, coperfusion experiments were done. The p38 MAPK and JNK2/3 inhibitor SB203580 partially protected FAT when coperfused with 10 nM gp120 (a). Similarly, both the specific JNK inhibitor SP600125 (b) and the specific p38 MAPK inhibitor MW069A (069 A) (c) partially protected FAT, suggesting that both p38 MAPK and JNK may be activated by gp120. Box plots were generated to compare each treatment (d). ANOVA and post hoc Tukey-Kramer test were used on pooled values from 30- to 50 min time points to determine statistical significance. ^#^*p* > .05 compared with buffer control, denoting complete protection. *p < .05 compared with both buffer control and to 10 nM gp120, denoting partial protection.

**Figure 4.**
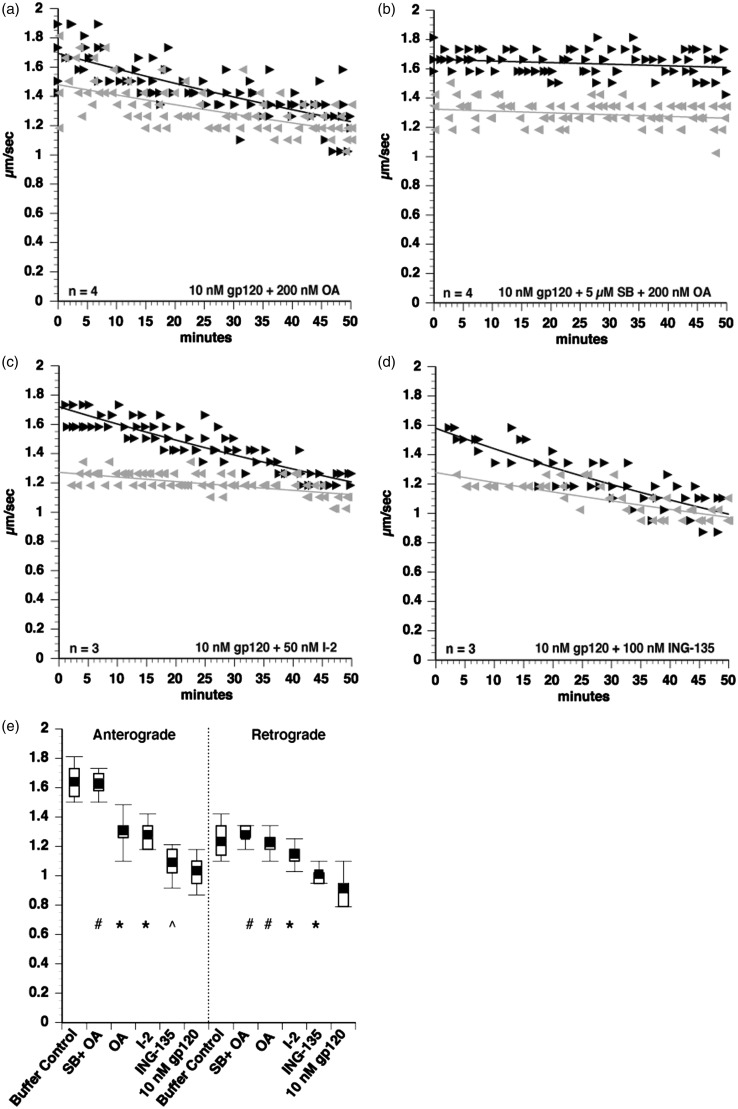
Gp120-induced inhibition of FAT involves activation of PP1 phosphatase. Since p38 MAPK and JNK inhibition both provided only partial protection, additional phosphotransferase pathways that affect FAT were examined through coperfusion of axoplasms with gp120 and appropriate inhibitors. Okadaic acid (OA), a serine-threonine phosphatase inhibitor, partially protected FAT from gp120-induced FAT impairment (a). Coperfusion with both OA and SB203580 (which inhibits JNK3 and p38s) had an additive effect to completely protect FAT (b), suggesting that phosphatase activation by gp120 occurs in parallel to JNK and p38 MAPK activation. To narrow down the phosphatase activated by gp120, the specific inhibitor of PP1 I–2 was tested and found to partially protect FAT (c), indicating that PP1 partially mediates the effect of gp120 on FAT. Since PP1 is known to activate GSK-3β to inhibit FAT (Morfini et al., 2004; Kanaan et al., 2011), this pathway was tested using a GSK-3β inhibitor ING-135. However, ING-135 did not protect anterograde FAT with marginal protection in the retrograde direction (d). ANOVA and post hoc Tukey-Kramer test were used on pooled values from 30- to 50 min time points to determine statistical significance. ^#^*p* > .05 compared with buffer control, denoting complete protection from gp120 impairment of FAT. *p < .005 compared with both buffer control and to 10 nM gp120, denoting partial protection from gp120 impairment of FAT. *p* > .05 compared with 10 nM gp120, denoting lack of protection from gp120 impairment of FAT.

**Figure 5.**
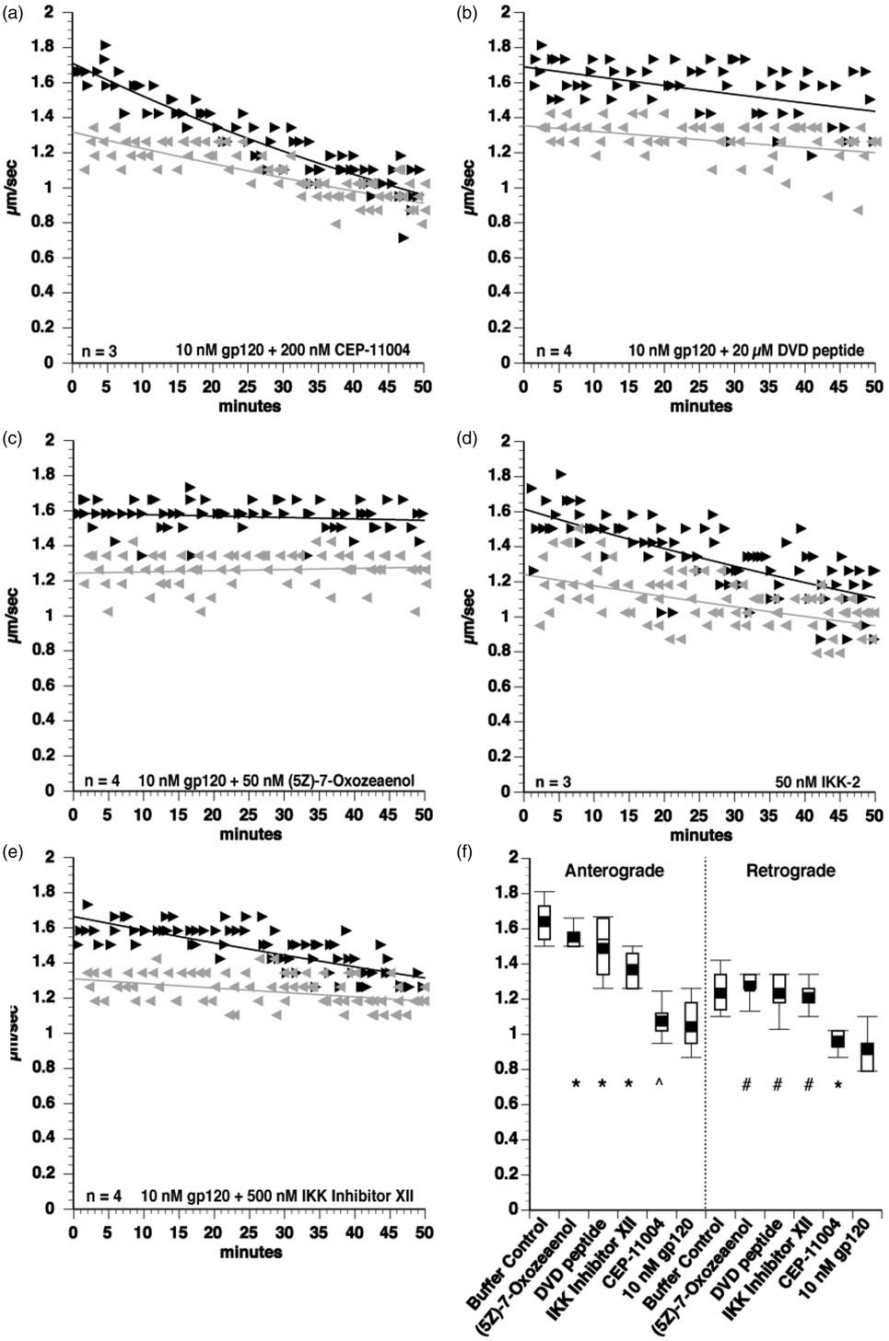
Gp120 activates the MAP3K Tak1 to impair FAT. To determine upstream activators of JNK and p38 MAPK mediating the effects of gp120 on FAT, coperfusion experiments were done using various MAP3K inhibitors. The MLK inhibitor CEP-11004 did not protect FAT from gp120 impairment (a), indicating that gp120 does not activate p38s and JNKs through an MLK-dependent pathway. However, the DVD peptide, which blocks conserved docking domains of a subset of MAP3Ks, did partially protect the toxic effect of gp120 on FAT (b), demonstrating that one or more DVD peptide-sensitive MAP3Ks mediate p38 MAPK and JNK activation by gp120. Extending these findings, the specific Tak1 MAP3K inhibitor (5 Z)-7-Oxozeaenol protected FAT in coperfusion experiments with gp120 (c). Since the Tak1 inhibitor showed strong protection, it was postulated that the activation of PP1 is downstream of Tak1 activation and a kinase downstream of Tak1 that is regulated by PP1, IKK-2, was tested. Perfusion of IKK-2 into isolated axoplasm inhibited both anterograde and retrograde FAT (d). Further, coperfusion of axoplasms with gp120 and the selective inhibitor of IKK-2 (Inhibitor XII) partially protected FAT impairments (e), indicating a role for IKK activation in FAT inhibition by gp120. ANOVA and post hoc Tukey-Kramer test were used on pooled values from 30- to 50 min time points to determine statistical significance. ^#^*p* > .05 compared with buffer control, denoting complete protection. *p < .005 compared with both buffer control and to 10 nM gp120, denoting partial protection. *p* > .05 compared with 10 nM gp120, denoting lack of protection.

### Primary Rat DRG Neuron Cultures

DRGs were dissected from Sprague-Dawley rat embryos at gestation Day 15 (Charles River). Pooled DRGs were dissociated in 2.5% trypsin in 1 × Hank's balanced salt solution (HBSS) for 10 min in a 37 ℃ water bath, and then the tissue was triturated 10 times with a Pasteur pipet coated with 0.1% BSA in 1 × HBSS. The DRGs in trypsin solution were incubated for an additional 10 min in the 37 ℃ water bath followed by another round of trituration and repeated for a total of three rounds. Fetal bovine serum (10%) was added to the cell suspension prior to centrifugation and resuspended in unsupplemented neural basal (NB) medium (Invitrogen). The DRG neurons were counted and plated at 300 cells per channel, of the μ-Slide VI that contains a six-channel format (ibidi), previously coated with 0.5 mg/mL poly-L-lysine (Sigma #P1399) and 10 μg/mL mouse laminin (Invitrogen). The DRG neurons were initially plated in a NB media mix supplemented with glutamax, pen-strep, BSA, and B27 (Invitrogen). After 2 to 4 hr, 50% of the initial plating media was changed with the NB media mix plus nerve growth factor (Invitrogen), and the neuronal cultures were maintained under humidified atmosphere containing 5% CO_2_ at 37 ℃. After 24 hr *in vitro*, during a 50% media change, the DRG neurons were treated with 2 nM recombinant gp120 (ImmunoDiagnostics, Inc #1001), 2 nM heat-inactivated recombinant gp120 (gp120 incubated for 1 hr in 37 ℃ water bath), or 2 nM recombinant gp120 + 15 nM (5 Z)-7-oxozeaenol (EMD Millipore #499610) an inhibitor of Tak1, or remained untreated as the control.

### Primary DRG Neurite Outgrowth Assays

At 72 hr *in vitro* and 48 hr posttreatment, the DRG neurons were fixed with a solution containing 10% paraformaldehyde, 1 × PBS, and 8% glutaraldehyde for 20 min at room temperature and then washed with 1 × HBSS three times for 5 min each. The DRG neurons were labeled via fluorescent immunocytochemistry of α-tubulin for neurite outgrowth measurements. The fixed DRG neurons were quenched for 5 min with 50 nM NH_4_Cl in 1 × PBS to reduce background, washed in 1 × PBS, permeabilized with 0.1% triton for 10 min, washed in 1 × PBS, blocked with 5% milk in 1 × PBS for 1 hr, incubated with DM1a α-tubulin primary antibody (Sigma #T9026) at 1:1000 in 0.1% triton-PBS overnight at 4 ℃, washed in 1 × PBS, incubated with AF488-conjugated secondary antibody (Invitrogen #A11029) at 1:400 in 0.1% triton-PBS for 1 hr at room temperature, and washed in 1 × PBS, and Vectashield anti-fade mounting media (Vector Laboratories) was applied to each chamber channel. Each well in the ibidi chambers was imaged in a series of micrographs that were tiled to allow visualization of the full extent of all neurites in a well for measurement by fluorescent confocal microscopy (Zeiss LSM 710; Zeiss 10 ×/0,30 Plan-NEOFLUAR). The images were collected and coded by one investigator and subsequently analyzed by another investigator blinded to the experimental condition. The results include the average percentage of DRG neurons, defined by a neuronal cell body with neurite extensions, the average percentage of DRG degenerating neurons, defined by a neuronal cell body with no neurite extensions, and average neurite outgrowth lengths per DRG neuron, using Zeiss Zen Lite imaging analysis software with the measurement module spline tracing feature. Three independent experiments were conducted for each condition, and the quantitative results were averaged for each group and presented as the mean ± *SEM*. Statistical significance was determined at *p* < .05 using one-way ANOVA with Bonferroni post hoc analysis.

## Results

### CXCR4 Involvement in the Activation of p38 MAPK by gp120

Previous studies of DSP indicated that gp120 binding to its coreceptor CXCR4 activates signaling cascades, including ones leading to activation of JNK and p38 MAPK (Bodner et al., 2004; Wilkerson et al., 2012). The interpretation has been that gp120 binding activates the G-protein coupled receptor CXCR4, causing activation of signaling cascades (Oh et al., 2001). To further explore the mechanism of p38 MAPK activation, we evaluated kinase activity in F11 cells differentiated with dibutryl-cAMP, which are rat DRG neurons hybridized with mouse neuroblastoma cells (Ghil et al., 2000). F11 cells were selected for these experiments since their ability to proliferate and differentiate avoids heterogeneity of neuronal cell types characteristic of DRG primary cultures, while retaining similar characteristics to DRG neurons (Francel et al., 1987; Shastry et al., 2001). A time course was performed in which differentiated F11 cells were treated with 5 nM gp120 for various times. It was found that gp120 transiently activated p38 MAPK 5 min after gp120 treatment, with subsequent deactivation at the later time points of 15 and 30 min (Figure 1(a)). To examine the role of CXCR4 activation on this effect, differentiated F11 cells were pretreated for 1 hr with 2 µM AMD3100, a small molecule bicyclam that inhibits binding of gp120 to CXCR4 (De Clercq et al., 1994; Donzella et al., 1998), then with 5 nM gp120. As expected, AMD3100 pretreatment abolished the early activation of p38 MAPK observed 5 min after gp120, demonstrating that transient activation of p38 MAPK depends upon binding of gp120 to CXCR4 (Figure 1). However, activation of p38 MAPK after 30 min of gp120 treatment was increased in AMD3100-pretreated cells (Figure 1), indicating gp120 treatment might also increase p38 activity though a CXCR4-independent pathway. This observation was consistent with our prior studies showing multiple pathways of gp120 internalization (Berth et al., 2015). Analysis with student's two-tailed *t* test (Figure 1(b)) confirmed the activation of p38 MAPK at 5 min after gp120 treatment compared with treatment with the diluent PBS (*p* < .05), the abrogation of p38 MAPK activation by gp120 treatment after AMD3100 pretreatment (*p* < .0001), and the activation of p38 MAPK after 30 min of AMD3100 and gp120 treatment compared with 5 min of AMD3100 and gp120 treatment (*p* < .05).

### Gp120 Impairs FAT in Isolated Squid Axoplasm

It has previously been demonstrated that a fraction of gp120 can be internalized independent of binding to CXCR4 (Berth et al., 2015). Accordingly, AMD3100 treatment of F11 cells prevented activation of p38 MAPK by gp120 treatment after 5 min, but not 30 min, suggesting that intracellular gp120 that had been internalized in a pathway independent of CXCR4 (Berth et al., 2015) might be responsible for kinase activation. Because DSP exhibits the same pattern of dying-back neuropathy observed in several unrelated dysferopathies (Morfini et al 2007), we tested whether intracellular gp120 might also cause dysregulation of FAT.

The isolated squid axoplasm model has proven instrumental in defining signaling pathways that regulate both anterograde (kinesin-1 dependent) and retrograde (cytoplasmic-dynein-dependent) FAT, a cellular process critical for maintenance of the axonal compartment (Brady, 1985; Brady et al., 1985; Brady et al., 1993; Song and Brady, 2013). Extruded axoplasms were placed on a coverslip with glass spacers, creating a perfusion chamber that allows for perfusion of various proteins and inhibitors. Then, video-enhanced contrast differential interference microscopy was performed, and FAT rate values obtained using a calibrated cursor (Song et al., 2016).

Perfusion of 10 nM gp120 profoundly impaired both anterograde and retrograde FAT in isolated axoplasms (Figure 2(b)). In contrast, heat-inactivated gp120 had no effect on FAT and was comparable to FAT in axoplasms perfused with buffer alone (not shown), suggesting that the toxic effect of gp120 depends on its conformation and biological activity (Figure 2(a)). Statistical comparison between 10 nM gp120 and 10 nM heat-inactivated gp120 revealed a significant difference between both anterograde and retrograde FAT rates (Figure 2(e)). Moreover, FAT was inhibited in both directions when gp120 was perfused at 100 pM, a more physiologically relevant concentration (Figure 2(c) and (e)). Some studies suggested that neurotoxicity differs between gp120 strains that preferentially bind the coreceptors CXCR4 and CCR5 (Bachis et al., 2009; Bachis et al., 2010). Based on these precedents, we compared inhibition of FAT between gp120 IIIB, which binds to CXCR4, and gp120 BaL, a CCR5-preferring strain. Demonstrating that inhibition of FAT is conserved across gp120 strains, both 10 nM gp120 BaL and 10 nM gp120 IIIB produced similar impairments of FAT (Figure 2(d) and (e)) compared with heat-inactivated gp120. Statistical analysis with the student's two-tailed *t* test demonstrated that perfusing axoplasms with 10 nM gp120 IIIB, 10 nM gp120 BaL, or 100 pM gp120 IIIB all significantly decreased anterograde and retrograde FAT compared with heat-inactivated gp120 (Figure 2(e)).

### Gp120 Activates p38 MAPK and JNK to Impair FAT

Previous work established that FAT is regulated by phosphotransferases (Morfini et al., 2002; Morfini et al., 2006; Morfini, Burns, et al., 2009). To identify kinases mediating the inhibitory effect of gp120 on FAT, 10 nM gp120 IIIB was coperfused with various inhibitors of kinases and phosphatases known to modulate FAT. Effectiveness of each inhibitor was evaluated using quantitative analysis with ANOVA and post hoc Tukey-Kramer tests, by comparing to both buffer control and to 10 nM gp120. (Morfini et al., 2006; Morfini, You, et al., 2009; Pigino et al., 2009; Morfini et al., 2013). Inhibitors of the protein kinases CK1 and CK2 did not prevent the toxic effect of gp120 on FAT (data not shown). However, the p38 MAPK and JNK inhibitor SB203580 (5 µM) partially prevented the impairment of FAT (Figure 3(a)). Quantitative analysis indicated that this effect was statistically significant when compared with 10 nM gp120 alone (Figure 3(d)). Although retrograde FAT rates were restored to buffer control levels, anterograde FAT velocities were still significantly lower than those observed with buffer controls (Figure 3(d)). The JNK-specific inhibitor SP600125 was coperfused with 10 nM gp120 to test the involvement of JNK kinases (Figure 3(c)), or 10 µM of the specific p38 MAPK inhibitor MW01-2-069 A-SRM (MW069A; [Munoz et al., 2007] to evaluate the involvement of p38 kinases [Figure 3(c)]). Quantitative analysis revealed that coperfusion of each of these inhibitors with gp120 partially protected anterograde FAT, as evidenced by velocities significantly slower than buffer control and significantly higher than perfusion with 10 nM gp120 alone (Figure 3(d)). However, analysis of retrograde FAT demonstrated that while MW069A completely protected FAT, SP600125 showed partial protection. These observations suggested a more prominent role of p38s than JNKs on the inhibitory effects elicited by gp120 perfusion. Collectively, these data suggested that gp120 activates signaling pathways involving both p38 MAPK and JNK to impair FAT.

### Gp120 Activates PP1 to Impair FAT

The observation that p38 MAPK and JNK inhibitors partially blocked toxic effects of gp120 on FAT suggested that gp120 might activate multiple regulatory pathways for FAT, prompting us to test additional inhibitors. Interestingly, the decrement of FAT caused by gp120 IIIB was also reduced by the addition of 200 nM okadaic acid, an inhibitor of serine or threonine phosphatases PP1, PP2A, and PP2B (IC50: 10 nM; Figure 4(a)). Quantitative analysis revealed partial protection of FAT in the anterograde direction and complete protection in the retrograde direction (Figure 4(e)). To test whether the activation of serine threonine phosphatases occurred in parallel to the activation of p38 MAPK and JNK, 10 nM gp120 IIIB was coperfused with both SB203580 and okadaic acid (Figure 4(b)). Significantly, this treatment completely rescued the impairment of both anterograde and retrograde FAT caused by gp120 IIIB (Figure 4(e)).

To narrow down which okadaic acid-sensitive phosphatase was activated by gp120 to inhibit FAT, 50 nM of inhibitor I-2 (a PP1-specific inhibitor) was coperfused with 10 nM gp120 IIIB (Figure 4(c)). Coperfusion of squid axoplasm with gp120 and I-2 partially protected FAT in both the anterograde and retrograde directions (Figure 4(e)). Since it has previously been shown that PP1 can impair FAT by activating GSK3β (Morfini et al., 2004), we coperfused axoplasm with gp120 and 100 nM of the specific GSK3β inhibitor ING-135. However, ING-135 did not prevent the inhibition of FAT elicited by gp120 (Figure 4(d) and (e)). Together, results from these experiments indicate that gp120 independently activates at least two signaling pathways in axons with the downstream targets p38 MAPK, JNK, and another one involving PP1, both contributing to its toxic effect on FAT.

### Gp120 Activates a Tak1 Pathway to Impair FAT

JNK3 and p38 kinases are activated by upstream MAPK kinases (MAP2Ks), which in turn are activated by upstream MAP kinase kinases (MAP3Ks; Gallo and Johnson, 2002), prompting us to determine whether specific MAP3Ks mediate the inhibitory effect of gp120 on FAT. First, an involvement of mixed lineage kinases (MLKs) was tested in coperfusion experiments using the pharmacological inhibitor CEP-11004 (Gallo and Johnson, 2002; Kim et al., 2004). Coperfusing 10 nM gp120 with 200 nM CEP-11004 did not prevent the inhibition of anterograde FAT by gp120 (Figure 5(a) and (f)) and only showed marginal protection of retrograde FAT, suggesting the pathway activated by gp120 to impair FAT does not include activation of MLKs.

MAP3Ks other than MLKs, which can also activate both p38 and JNK include Tak1 and Ask1 (Gallo and Johnson, 2002). To determine whether other MAP3Ks mediated gp120-induced impairment of FAT, additional inhibitors were evaluated in coperfusion experiments. First, gp120 was coperfused with the DVD peptide, a peptide that competes with docking of MAP2Ks to a subset of MAP3Ks (Takekawa et al., 2005). Interestingly, DVD peptide partially protected the inhibition of anterograde FAT and completely protected gp120-induced inhibition of retrograde FAT (Figure 5(b)). Extending these findings, the selective Tak1 inhibitor (5 Z)-7-Oxozeaenol (50 nM) completely prevented the effects of gp120 in coperfusion experiments (Figure 5(c)), identifying Tak1 as an upstream MAP3K responsible for gp120-induced impairment of FAT. Although ANOVA analysis only showed partial protection of anterograde FAT, further analysis using the Spearman's correlation indicated that FAT did not decline over time.

Since the Tak1 inhibitor (5 Z)-7-Oxozeaenol completely protected FAT, activation of PP1 is also likely to occur in a pathway downstream of Tak1. Interestingly, Tak1 was shown to interact with PP1 (Platholi et al., 2014), and PP1 mediates activation of the inhibitor of IKK complex by Tak1, which leads to IKK-2-induced activation of nuclear factor kappa-light-chain-enhancer of activated B cells (NF-κB; Mitsuhashi et al., 2008). To determine whether this pathway contributed to inhibition of FAT by gp120, recombinant IKK-2 (Signal Chem) was perfused into axoplasm. IKK-2 inhibited both anterograde and retrograde FAT (Figure 5(d)), revealing a novel effect of IKK-2 on FAT. In addition, coperfusion of gp120 with the IKK-2 inhibitor XII resulted in a partial protection of anterograde FAT and a complete protection of retrograde FAT (Figure 5(e) and (f)), demonstrating that IKK-2 is a downstream target of Tak1 to impair FAT. Taken together, experiments using the squid axoplasm preparation indicate that intra-axonal gp120 causes dysregulation of FAT by promoting abnormal activation of Tak 1, which in turn promotes activation of multiple signaling cascades comprising specific kinases and phosphatases.

### Gp120 Activation of a Tak1 Pathway Also Impairs Sensory Neuron Neurite Outgrowth

**Figure 6.**
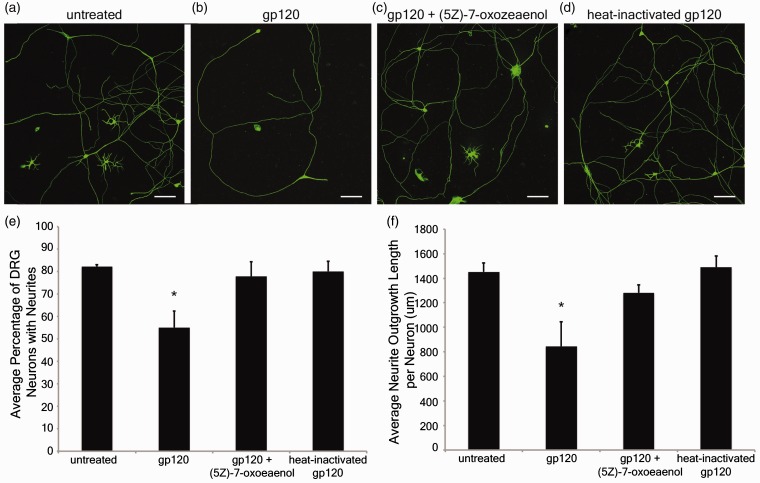
Gp120 impairs neurite outgrowth through a Tak1-dependent mechanism. Primary rat dorsal root ganglion (DRG) neurons were treated with gp120 to determine whether gp120 altered neuronal morphology *via* the ability to grow or maintain neurite extensions. Representative photomicrographs illustrate DRG neurons that were untreated (a), treated with 2 nM gp120 (b), 2 nM gp120 plus 15 nM (5 Z)-7-oxozeaenol (c), or 2 nM heat-inactivated gp120 (d). Scale bars indicate 100 µm. The average percentage of DRG neurons exhibiting neurite extensions (e); calculated from separating the DRG neurons into two categories, one included counts of DRG neurons that displayed neurites and the other included counts of DRG neurons that lacked neurites. The average neurite outgrowth length per DRG neuron was determined by measuring all neurites within the sample and dividing by the number of DRG neurons that displayed neurites (f), results given in micrometers. All results averaged from three independent experiments that examined 3,154 neurons in total, including 2,374 neurons exhibiting neurites and 780 neuronal cell bodies without neurites. *p < .05 compared with untreated control, gp120 + (5 Z)-7-oxozeaenol, and heat-inactivated gp120 groups.

Results from squid axoplasm experiments indicated that gp120-induced impairment of FAT is mediated by Tak1 activation. To evaluate the relevance of these findings to DSP, the potential for gp120 to affect neurites projecting from primary sensory neurons *via* Tak1 was assessed. Neurite outgrowth measurements were made on DRG cultured neurons prepared from E15 rat embryos. Representative photomicrographs are presented in Figure 6 of primary DRG neurons that were left untreated (Figure 6(a)) or treated with 2 nM gp120 (Figure 6(b)), 2nM gp120 + 15 nM (5 Z)-7-oxozeaenol (Tak1 inhibitor; Figure 6(c)), or 2 nM heat-inactivated gp120 (Figure 6(d)). The treatment was initiated 24 hr after plating (1 day *in vitro*), and all groups were fixed at 48 hr posttreatment (3 days *in vitro*) and immunostained with DM1A, primary antibody specific to α-tubulin for analysis of neurite outgrowth.

**Figure 7.**
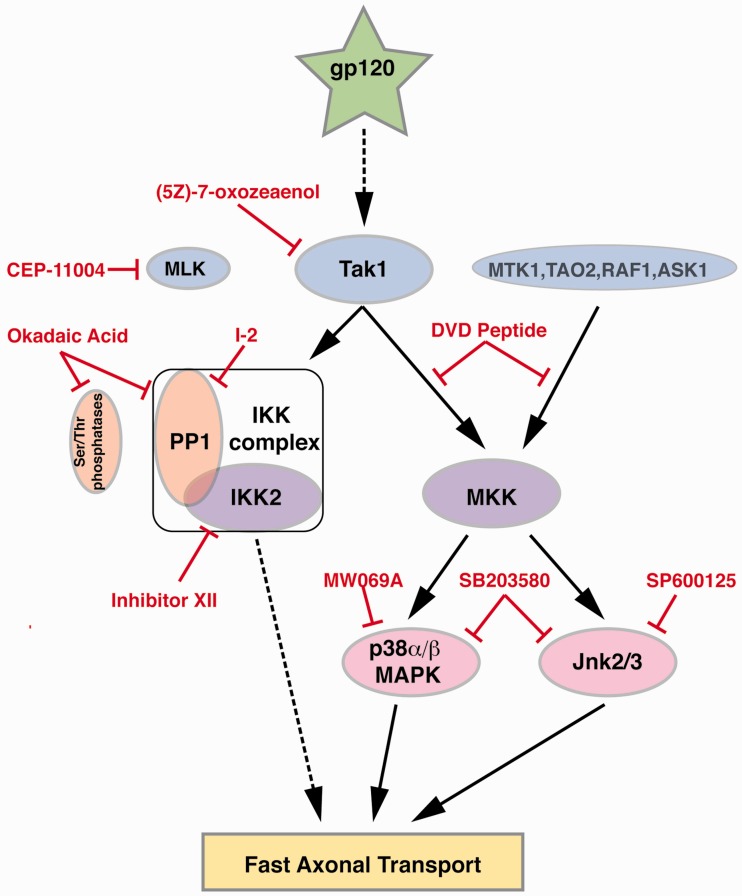
Diagram depicting kinase pathways activated by gp120. The use of pharmacological inhibitors in axonal transport studies here delineated novel signaling pathways by which gp120 impairs FAT. Gp120 was found to activate, directly or indirectly (dotted line) the MAP3K Tak1. Tak1 in turn activates at least two independent signaling pathways. In one pathway, Tak1 activates the IKK complex through a mechanism that requires PP1 activity and leads to activation of IKK-2. How IKK-2 inhibits FAT is currently unknown and depicted by the dotted line. In the second pathway, Tak1 activates canonical MAP2Ks which in turn, promote activation of both p38 MAPK and JNK. Both p38 MAPK and JNK are known to phosphorylate kinesin to inhibit FAT. (5 Z)-7-Oxozeaenol: Tak-1 inhibitor. DVD peptide blocks conserved docking domains to inhibit certain MAP3K's. Okadaic acid inhibits serine or threonine phosphatases. I-2: specific inhibitor of the serine or threonine phosphatase PP1. Inhibitor XII: a specific inhibitor of IKK-2. MW069A: a specific inhibitor of p38 MAPK. SP600125: a specific inhibitor of JNK. SB203580: an inhibitor of p38 MAPK and JNK3.

DRG neurons were separated into two categories according to their morphology, those that had neurites extending from the perikarya and those that did not have neurite extensions. Extracellular treatment with gp120 resulted in a significant reduction in the percentage of DRG neurons that exhibited neurite extensions (55 ± 7%; *p* < .05), compared with untreated DRG neurons (82 ± 0.8%; Figure 6(a), (b), and (e)). Examination of neurite outgrowth measurements revealed that treatment with gp120 also resulted in a significant reduction in the average neurite outgrowth length per DRG neuron (845 ± 199 µm/DRG neuron; *p* < .05), compared with untreated DRG neurons (1452 ± 73 µm/DRG neuron; Figure 6(a), (b), and (f)). However, cotreatment with gp120 + (5 Z)-7-oxozeaenol demonstrated a protective effect by significantly increasing both the percentage of DRG neurons exhibiting neurite extensions (78 ± 7%; Figure 6(a), (c), and (e)) and the average neurite outgrowth length per DRG neuron (1281 ± 67 µm/DRG neuron; Figure 6(a), (c), and (f)), compared with DRG neurons with gp120 alone. DRG neurons treated with heat-inactivated gp120 did not differ significantly from untreated controls for neurite extensions (80 ± 4%; Figure 6(a), (d), and (e)) or for the average neurite outgrowth length per DRG neuron (1490 ± 92 µm/DRG neuron; Figure 6(a), (d), and (f)), compared with untreated DRG neurons. These results suggest that gp120 reduces the ability of DRG sensory neurons to maintain neurite extensions, and gp120's negative impact on neurite outgrowth and sensory neuron morphology can be reversed by a Tak1-specific inhibitor. Together, these results are consistent with results obtained with the isolated axoplasm preparation, providing further evidence for a link between gp120-mediated Tak1 activation and neurite degeneration.

## Discussion

Although DSP is the most prevalent neurological complication of HIV, affecting close to 30% of patients, its pathogenesis remains unclear and treatments are limited (Phillips et al., 2010). Proposed pathological mechanisms of DSP include indirect neurotoxicity by activation of macrophages and direct neurotoxicity by release of viral proteins, such as gp120, which interact with DRG neurons. A recent study indicated that gp120 is internalized by DRG neurons and localizes within their axons (Berth et al., 2015), suggesting the possibility of an intra-neuronal mechanism for gp120 to cause neurotoxicity.

Several studies utilizing transgenic models or gp120 treatments have linked gp120 to axonal degeneration (Herzberg and Sagen, 2001; Michaud et al., 2001; Keswani et al., 2006; Melli et al., 2006; Robinson et al., 2007), but the underlying mechanisms underlying for this critical pathogenic event in DSP remained unclear. Induction of neuronal apoptosis has been the most commonly studied parameter for gp120 neurotoxicity (Zheng et al., 1999; Bachis et al., 2003; Bodner et al., 2004; Singh et al., 2004; Singh et al., 2005),. However, apoptosis is not a major feature of DSP, and targeting cell death has not been effective at ameliorating disease in dying-back neuropathies (Sagot et al., 1997; Chiesa et al., 2005; Gould et al., 2006), Instead, the pattern of sensory neuron degeneration in DSP progresses distally to proximally in a “stocking and glove” fashion (Keswani et al., 2002), placing DSP in the category of dying-back neuropathies where degeneration represents an early pathogenic feature that long precedes cell death.

How might intra-neuronal gp120 cause the axonal degeneration phenotype seen in DSP? One possible mechanism is through impairments in FAT. Loss-of-function mutations of the motor proteins kinesin or CDyn are sufficient to cause dying-back neuropathies (Reid et al., 2002; Puls et al., 2003; Munch et al., 2005; Ebbing et al., 2008; Dupuis et al., 2009). Since the concentration of kinesin is 0.5 µM and tubulin is 25 µM in squid axoplasm (Morris and Lasek, 1984; Brady et al., 1990), the concentration of perfused gp120 was not high enough to cause sequestration of kinesin or impair FAT through binding to microtubules (Brady et al., 1990). Further, gp120 perfusion did not alter microtubule structure in the squid axoplasm (data not shown). Therefore, a more plausible explanation is that the severe effect on FAT elicited by gp120 results from activation of signaling cascades that regulate FAT.

Abnormal activation of signaling pathways for regulation of FAT has been implicated in a multiple dying-back neuropathies, including Alzheimer's disease (Pigino et al., 2009; Kanaan et al., 2012), Parkinson's disease (Morfini et al., 2007; Chu et al., 2012), amyotrophic lateral sclerosis (Bosco et al., 2010; Morfini et al., 2013), spinal bulbar muscular atrophy (Morfini et al., 2006), and Huntington's disease (Szebenyi et al., 2003; Morfini, You, et al., 2009), leading to the coinage of “dysferopathies” as a term to describe dying-back neuropathies in which impaired FAT has a prominent role (Morfini et al., 2007). To examine whether DSP itself might be a dysferopathy, the ability of gp120 to alter the activity of kinases involved in regulation of FAT was assessed. Previously, gp120 treatment had been reported to activate JNK (Bodner et al., 2004; Khan et al., 2004) or p38 MAPK (Hu et al., 2005; Singh et al., 2005; Medders et al., 2010) in neurons. The assumption in these studies was that kinase activation and neurotoxic effects of gp120 were dependent upon binding to its coreceptor CXCR4, a GPCR that promotes a transient activation of kinase signaling cascades. Sustained activation of either JNK or P38 MAPK can affect FAT (Morfini et al., 2006; Morfini, You, et al., 2009; Bosco et al., 2010; Morfini et al., 2013), but transient activation is unlikely to significantly affect transport. However, elimination of the CXCR4 response with AMD3100 pretreatment unmasked a slower activation of p38 MAPK after 30 min of gp120 exposure.

How might this phosphorylation of p38 MAPK occur with no CXCR4 activation? Given that a portion of gp120 internalization in F11 cells was independent of CXCR4 binding (Berth et al., 2015), the delayed activation of p38 MAPK, corresponding to the timing of gp120 internalization, could be due to the activation of signaling cascades by intracellular gp120. This delayed activation of P38 MAPK would be masked by activation through CXCR4.

The neurotoxicity of different gp120 strains has been a matter of dispute. Some past studies found that T-tropic gp120 was more neurotoxic than M-tropic gp120 (Zheng et al., 1999; Frost et al., 2009; Bachis et al., 2010), but other studies found similar neurotoxicity for T-tropic and M-tropic gp120 strains (Yi et al., 2004; Kaul et al., 2007). The divergent strains T-tropic gp120 IIIB (CXCR4) and the M-tropic gp120 BaL (CXCR5) were comparable in their ability to inhibit FAT in axoplasm, suggesting that the effect on FAT is likely to be conserved across strains, even though some studies reported a neuroprotective effect of CCR5 (Kaul et al., 2007). An explanation for similar neurotoxic effects between the two strains is that squid axoplasm is a plasma membrane-free preparation, so any effects of gp120 seen in these experiments are independent of coreceptor activation.

Since the amount of free gp120 in plasma has been determined to be in the low picomolar to low nanomolar range (Gilbert et al., 1991; Oh et al., 1992), 100 pM gp120 was perfused to determine if a lower, more physiological relevant concentration might affect transport. In fact, 100 pM gp120 impaired FAT to a similar extent as 10 nM gp120. The local concentration of gp120 around DRG neurons has not been directly measured but is likely higher than plasma concentrations due to the close proximity of infected cells binding to extracellular matrix components or local glial swelling (Nath, 2002; Krathwohl and Kaiser, 2004). An important point is that DSP takes decades to develop, although subclinical signs are commonly manifested in HIV patients. Thus, smaller amounts of gp120 internalized by DRG neurons could gradually compromise neuronal function over decades by compromising FAT in DRG neurons.

Partial protection of FAT from the effects of gp120 with coperfusion of axoplasm with gp120 and various inhibitors of kinases and phosphatases identified various components of the signaling pathways likely to mediate gp120 effects on FAT. Partial protection by inhibitors of p38 MAPK, JNK, and PP1 (Figures 3 and 4) suggested that gp120 activates p38 MAPK, JNK, and PP1 to inhibit FAT. In contrast, an inhibitor of TAK1 offered full protection of FAT from the action of gp120 (Figure 5(c)), suggesting a critical role for this MAP3K in the signaling pathways associated with DSP. The additive effect of MAPK and PP1 inhibitors indicated that PP1 activation occurred in an independent, parallel pathway to the one responsible for activation of p38 MAPK and JNK, both of which may be linked to TAK1.

Additional evidence that gp120 impairs neuronal function by activation of Tak1 was provided by analyzing primary DRG neuron neurite extension after exposure to gp120 *in vitro*. Exposing primary cultured rat DRG neurons to gp120 directly affected the outgrowth of neurites, through either inhibition of neurite sprouting or initiating degeneration of previously extended neurites. The deleterious effect of gp120 on DRG neurite outgrowth was reversed by concurrent treatment with the Tak1 inhibitor, (5 Z)-7-oxozeaenol, suggesting that DRG neurite susceptibility to gp120 exposure is mediated through Tak1 signaling.

Tak1 MAP3K is well established as an upstream MAP3K for p38 MAPK and JNK (Gallo and Johnson, 2002), but inhibiting Tak1 also provided complete protection of FAT, suggesting that activation of PP1 is downstream of Tak1. Since Tak1 is known to activate IKK-2 (Sakurai, 2012), and PP1 has been found to interact with the IKK complex to positively regulate IKK-2 activation (Mitsuhashi et al., 2008), the activation of IKK-2 by gp120 was tested. In fact, recombinant IKK-2 inhibited anterograde and retrograde FAT, and coperfusion of axoplasm with both gp120 and the IKK-2 inhibitor XII exhibited partial protection, indicating that gp120 activates IKK-2 to inhibit FAT. It is unclear if PP1 is activated by gp120, or if PP1 activity is required for Tak1 activation of IKK-2. These results define a novel role for IKK-2 in regulation of FAT, which is outside of its role in activating NF-κB. Indeed, accumulations of both IKK-2 and its phosphorylated downstream target IκBα have been detected in axons, primarily in initial segments and nodes of Ranvier extending into paranodes (Schultz et al., 2006; Politi et al., 2008). Phosphorylated IκBα associates with microtubules (Politi et al., 2008), placing it in an appropriate location to regulate FAT. IκBα was also reported in association with dynein light chain through a yeast two-hybrid screen, coimmunoprecipitation in cell lines and colocalization in immunofluorescence (Crepieux et al., 1997). However, the mechanism by which IKK-2 or IκBα regulates FAT remains to be defined. Future experiments will determine whether IKK-2 or IκBα might directly phosphorylate kinesin or dynein, or if they activate an intermediate kinase that phosphorylates either motor protein to regulate FAT.

Collectively, results from experiments here revealed a novel mechanism of neurotoxicity for gp120. Gp120 is internalized by neurons, then localized and transported in their axons (Berth et al., 2015). Intra-axonal gp120 inhibits FAT by affecting regulatory signaling cascades. As impaired FAT promotes axonal pathology, observations here are consistent with the dying-back neurodegeneration seen in DSP. Indeed, pharmacological inhibition of the signaling pathway through which gp120 inhibits FAT in isolated axoplasm was sufficient to prevent neuritic abnormalities in mammalian DRG neurons. This direct effect of gp120 does not exclude the possibility of indirect actions, and it is likely that multiple actions of gp120 synergize to harm DRG neurons. For example, the activation of CXCR4 by gp120 that leads to p38 MAPK activation might synergize with p38 MAPK activation caused by intra-axonal gp120. Additionally, TNF-α has been strongly implicated in the inflammatory response in the indirect pathway for DSP (Herzberg and Sagen, 2001; Keswani et al., 2003; Zheng et al., 2011). Intriguingly, TNF-α activates the NF-κB pathway through Tak1, which is the same pathway that we have found activated by intra-axonal gp120. These multiple mechanisms leading to activation of Tak1 could very well synergize with each other. Regardless, we propose that impairment of FAT by gp120 is a critical step in the pathway to the development of DSP, making DSP another member of the class of neurological diseases that can be considered dysferopathies. Promoting normal regulation of FAT may represent a promising avenue for therapeutic interventions for DSP.

## References

[bibr1-1759091416679073] Bachis A. Aden S. A. Nosheny R. L. Andrews P. M. Mocchetti I. (2006) Axonal transport of human immunodeficiency virus type 1 envelope protein glycoprotein 120 is found in association with neuronal apoptosis. The Journal of Neuroscience 26: 6771–6780. 16793884 10.1523/JNEUROSCI.1054-06.2006 PMC6673819

[bibr2-1759091416679073] Bachis A. Biggio F. Major E. O. Mocchetti I. (2009) M- and T-tropic HIVs promote apoptosis in rat neurons. Journal of Neuroimmune Pharmacology 4: 150–160. 19034668 10.1007/s11481-008-9141-3 PMC2683632

[bibr3-1759091416679073] Bachis A. Cruz M. I. Mocchetti I. (2010) M-tropic HIV envelope protein gp120 exhibits a different neuropathological profile than T-tropic gp120 in rat striatum. The European Journal of Neuroscience 32: 570–578. 20670282 10.1111/j.1460-9568.2010.07325.x PMC2924467

[bibr4-1759091416679073] Bachis A. Major E. O. Mocchetti I. (2003) Brain-derived neurotrophic factor inhibits human immunodeficiency virus-1/gp120-mediated cerebellar granule cell death by preventing gp120 internalization. The Journal of Neuroscience 23: 5715–5722. 12843275 10.1523/JNEUROSCI.23-13-05715.2003 PMC6741234

[bibr5-1759091416679073] Berth S. Caicedo H. H. Sarma T. Morfini G. Brady S. T. (2015) Internalization and axonal transport of the HIV glycoprotein gp120. ASN Neuro 7: 1–15. 10.1177/1759091414568186 PMC4720180 25636314

[bibr6-1759091416679073] Bodner A. Toth P. T. Miller R. J. (2004) Activation of c-Jun N-terminal kinase mediates gp120IIIB- and nucleoside analogue-induced sensory neuron toxicity. Experimental Neurology 188: 246–253. 15246824 10.1016/j.expneurol.2004.04.009

[bibr7-1759091416679073] Bosco D. A. Morfini G. Karabacak N. M. Song Y. Gros-Louis F. Pasinelli P. Brown R. H. Jr. (2010) Wild-type and mutant SOD1 share an aberrant conformation and a common pathogenic pathway in ALS. Nature Neuroscience 13: 1396–1403. 20953194 10.1038/nn.2660 PMC2967729

[bibr8-1759091416679073] Brady S. T. (1985) A novel brain ATPase with properties expected for the fast axonal transport motor. Nature 317: 73–75. 2412134 10.1038/317073a0

[bibr9-1759091416679073] Brady S. T. Lasek R. J. Allen R. D. (1985) Video microscopy of fast axonal transport in extruded axoplasm: A new model for study of molecular mechanisms. Cell Motility 5: 81–101. 2580632 10.1002/cm.970050203

[bibr10-1759091416679073] Brady S. T. Pfister K. K. Bloom G. S. (1990) A monoclonal antibody against kinesin inhibits both anterograde and retrograde fast axonal transport in squid axoplasm. Proceedings of the National Academy of Sciences of the United States of America 87: 1061–1065. 1689058 10.1073/pnas.87.3.1061 PMC53410

[bibr11-1759091416679073] Brady S. T. Richards B. W. Leopold P. L. (1993) Assay of vesicle motility in squid axoplasm. Methods in Cell Biology 39: 191–202. 7504159 10.1016/s0091-679x(08)60171-5

[bibr12-1759091416679073] Brenneman D. E. Westbrook G. L. Fitzgerald S. P. Ennist D. L. Elkins K. L. Ruff M. R. Pert C. B. (1988) Neuronal cell killing by the envelope protein of HIV and its prevention by vasoactive intestinal peptide. Nature 335: 639–642. 2845276 10.1038/335639a0

[bibr13-1759091416679073] Chiesa R. Piccardo P. Dossena S. Nowoslawski L. Roth K. A. Ghetti B. Harris D. A. (2005) Bax deletion prevents neuronal loss but not neurological symptoms in a transgenic model of inherited prion disease. Proceedings of the National Academy of Sciences of the United States of America 102: 238–243. 15618403 10.1073/pnas.0406173102 PMC544044

[bibr14-1759091416679073] Chu Y. Morfini G. A. Langhamer L. B. He Y. Brady S. T. Kordower J. H. (2012) Alterations in axonal transport motor proteins in sporadic and experimental Parkinson's disease. Brain 135: 2058–2073. 22719003 10.1093/brain/aws133 PMC4571141

[bibr15-1759091416679073] Crepieux P. Kwon H. Leclerc N. Spencer W. Richard S. Lin R. Hiscott J. (1997) I kappaB alpha physically interacts with a cytoskeleton-associated protein through its signal response domain. Molecular and Cellular Biology 17: 7375–7385. 9372968 10.1128/mcb.17.12.7375 PMC232593

[bibr16-1759091416679073] DeBoer S. R. You Y. Szodorai A. Kaminska A. Pigino G. Nwabuisi E. Morfini G. (2008) Conventional kinesin holoenzymes are composed of heavy and light chain homodimers. Biochemistry 47: 4535–4543. 18361505 10.1021/bi702445j PMC2644488

[bibr17-1759091416679073] De Clercq E. Yamamoto N. Pauwels R. Balzarini J. Witvrouw M. De Vreese K. Abrams M. (1994) Highly potent and selective inhibition of human immunodeficiency virus by the bicyclam derivative JM3100. Antimicrobial Agents and Chemotherapy 38: 668–674. 7913308 10.1128/aac.38.4.668 PMC284523

[bibr18-1759091416679073] Donzella G. A. Schols D. Lin S. W. Este J. A. Nagashima K. A. Maddon P. J. Moore J. P. (1998) AMD3100, a small molecule inhibitor of HIV-1 entry via the CXCR4 co-receptor. Nature Medicine 4: 72–77. 10.1038/nm0198-072 9427609

[bibr19-1759091416679073] Dorsey S. G. Morton P. G. (2006) HIV peripheral neuropathy: Pathophysiology and clinical implications. AACN Clinical Issues 17: 30–36. 16462406 10.1097/00044067-200601000-00004

[bibr20-1759091416679073] Dupuis L. Fergani A. Braunstein K. E. Eschbach J. Holl N. Rene F. Loeffler J. P. (2009) Mice with a mutation in the dynein heavy chain 1 gene display sensory neuropathy but lack motor neuron disease. Experimental Neurology 215: 146–152. 18952079 10.1016/j.expneurol.2008.09.019

[bibr21-1759091416679073] Ebbing B. Mann K. Starosta A. Jaud J. Schols L. Schule R. Woehlke G. (2008) Effect of spastic paraplegia mutations in KIF5A kinesin on transport activity. Human Molecular Genetics 17: 1245–1252. 18203753 10.1093/hmg/ddn014

[bibr22-1759091416679073] Francel P. C. Harris K. Smith M. Fishman M. C. Dawson G. Miller R. J. (1987) Neurochemical characteristics of a novel dorsal root ganglion X neuroblastoma hybrid cell line, F-11. Journal of Neurochemistry 48: 1624–1631. 2435852 10.1111/j.1471-4159.1987.tb05711.x

[bibr23-1759091416679073] Frost B. Jacks R. L. Diamond M. I. (2009) Propagation of tau misfolding from the outside to the inside of a cell. Journal of Neurochemistry 284: 12845–12852. 10.1074/jbc.M808759200 PMC2676015 19282288

[bibr24-1759091416679073] Gallo K. A. Johnson G. L. (2002) Mixed-lineage kinase control of JNK and p38 MAPK pathways. Nature Reviews. Molecular Cell Biology 3: 663–672. 12209126 10.1038/nrm906

[bibr25-1759091416679073] Ghil S. H. Kim B. J. Lee Y. D. Suh-Kim H. (2000) Neurite outgrowth induced by cyclic AMP can be modulated by the alpha subunit of Go. Journal of Neurochemistry 74: 151–158. 10617116 10.1046/j.1471-4159.2000.0740151.x

[bibr26-1759091416679073] Gilbert M. Kirihara J. Mills J. (1991) Enzyme-linked immunoassay for human immunodeficiency virus type 1 envelope glycoprotein 120. Journal of Clinical Microbiology 29: 142–147. 1993748 10.1128/jcm.29.1.142-147.1991 PMC269718

[bibr27-1759091416679073] Gould T. W. Buss R. R. Vinsant S. Prevette D. Sun W. Knudson C. M. Oppenheim R. W. (2006) Complete dissociation of motor neuron death from motor dysfunction by Bax deletion in a mouse model of ALS. The Journal of Neuroscience 26: 8774–8786. 16928866 10.1523/JNEUROSCI.2315-06.2006 PMC6674380

[bibr28-1759091416679073] Herzberg U. Sagen J. (2001) Peripheral nerve exposure to HIV viral envelope protein gp120 induces neuropathic pain and spinal gliosis. Journal of Neuroimmunology 116: 29–39. 11311327 10.1016/s0165-5728(01)00288-0

[bibr29-1759091416679073] Hesselgesser J. Taub D. Baskar P. Greenberg M. Hoxie J. Kolson D. L. Horuk R. (1998) Neuronal apoptosis induced by HIV-1 gp120 and the chemokine SDF-1 alpha is mediated by the chemokine receptor CXCR4. Current Biology 8: 595–598. 9601645 10.1016/s0960-9822(98)70230-1

[bibr30-1759091416679073] Hu S. Sheng W. S. Lokensgard J. R. Peterson P. K. (2005) Morphine potentiates HIV-1 gp120-induced neuronal apoptosis. The Journal of Infectious Diseases 191: 886–889. 15717263 10.1086/427830

[bibr131-1759091416679073] Kanaan, N. M., Morfini, G. A., Lapointe, N. E., Pigino, G. F., Patterson, K. R., Song, Y., … Binder, L. I. (2011). Pathogenic Forms of Tau Inhibit Kinesin-Dependent Axonal Transport through a Mechanism Involving Activation of Axonal Phosphotransferases. *The Journal of neuroscience: The official journal of the Society for Neuroscience*, *31*, 9858–9868. 10.1523/JNEUROSCI.0560-11.2011 PMC3391724 21734277

[bibr31-1759091416679073] Kanaan N. M. Morfini G. Pigino G. LaPointe N. E. Andreadis A. Song Y. Brady S. T. (2012) Phosphorylation in the amino terminus of tau prevents inhibition of anterograde axonal transport. Neurobiology of Aging 33: 826.e815–e830. 10.1016/j.neurobiolaging.2011.06.006 PMC3272324 21794954

[bibr32-1759091416679073] Kaul M. Ma Q. Medders K. E. Desai M. K. Lipton S. A. (2007) HIV-1 coreceptors CCR5 and CXCR4 both mediate neuronal cell death but CCR5 paradoxically can also contribute to protection. Cell Death and Differentiation 14: 296–305. 16841089 10.1038/sj.cdd.4402006

[bibr33-1759091416679073] Keswani S. C. Jack C. Zhou C. Hoke A. (2006) Establishment of a rodent model of HIV-associated sensory neuropathy. The Journal of Neuroscience 26: 10299–10304. 17021185 10.1523/JNEUROSCI.3135-06.2006 PMC6674617

[bibr34-1759091416679073] Keswani S. C. Pardo C. A. Cherry C. L. Hoke A. McArthur J. C. (2002) HIV-associated sensory neuropathies. Aids 16: 2105–2117. 12409731 10.1097/00002030-200211080-00002

[bibr35-1759091416679073] Keswani S. C. Polley M. Pardo C. A. Griffin J. W. McArthur J. C. Hoke A. (2003) Schwann cell chemokine receptors mediate HIV-1 gp120 toxicity to sensory neurons. Annals of Neurology 54: 287–296. 12953261 10.1002/ana.10645

[bibr36-1759091416679073] Khan M. Z. Brandimarti R. Patel J. P. Huynh N. Wang J. Huang Z. Meucci O. (2004) Apoptotic and antiapoptotic effects of CXCR4: Is it a matter of intrinsic efficacy? Implications for HIV neuropathogenesis. AIDS Research and Human Retroviruses 20: 1063–1071. 15585097 10.1089/aid.2004.20.1063 PMC2669736

[bibr37-1759091416679073] Kim K. Y. Kim B. C. Xu Z. Kim S. J. (2004) Mixed lineage kinase 3 (MLK3)-activated p38 MAP kinase mediates transforming growth factor-beta-induced apoptosis in hepatoma cells. Journal of Neurochemistry 279: 29478–29484. 10.1074/jbc.M313947200 15069087

[bibr38-1759091416679073] Kranick S. M. Nath A. (2012) Neurologic complications of HIV-1 infection and its treatment in the era of antiretroviral therapy. Continuum (Minneapolis, Minn.) 18: 1319–1337. 10.1212/01.CON.0000423849.24900.ec PMC3760534 23221843

[bibr39-1759091416679073] Krathwohl M. D. Kaiser J. L. (2004) HIV-1 promotes quiescence in human neural progenitor cells. The Journal of Infectious Diseases 190: 216–226. 15216454 10.1086/422008

[bibr40-1759091416679073] Lannuzel A. Lledo P. M. Lamghitnia H. O. Vincent J. D. Tardieu M. (1995) HIV-1 envelope proteins gp120 and gp160 potentiate NMDA-induced [Ca2+]i increase, alter [Ca2+]i homeostasis and induce neurotoxicity in human embryonic neurons. The European Journal of Neuroscience 7: 2285–2293. 8563977 10.1111/j.1460-9568.1995.tb00649.x

[bibr41-1759091416679073] Lehmann H. C. Chen W. Borzan J. Mankowski J. L. Hoke A. (2011) Mitochondrial dysfunction in distal axons contributes to human immunodeficiency virus sensory neuropathy. Annals of Neurology 69: 100–110. 21280080 10.1002/ana.22150 PMC3051401

[bibr42-1759091416679073] Medders K. E. Sejbuk N. E. Maung R. Desai M. K. Kaul M. (2010) Activation of p38 MAPK is required in monocytic and neuronal cells for HIV glycoprotein 120-induced neurotoxicity. Journal of Immunology 185: 4883–4895. 10.4049/jimmunol.0902535 PMC3848867 20855878

[bibr43-1759091416679073] Melli G. Keswani S. C. Fischer A. Chen W. Hoke A. (2006) Spatially distinct and functionally independent mechanisms of axonal degeneration in a model of HIV-associated sensory neuropathy. Brain 129: 1330–1338. 16537566 10.1093/brain/awl058

[bibr44-1759091416679073] Meucci O. Fatatis A. Simen A. A. Bushell T. J. Gray P. W. Miller R. J. (1998) Chemokines regulate hippocampal neuronal signaling and gp120 neurotoxicity. Proceedings of the National Academy of Sciences of the United States of America 95: 14500–14505. 9826729 10.1073/pnas.95.24.14500 PMC24402

[bibr45-1759091416679073] Meucci O. Miller R. J. (1996) gp120-induced neurotoxicity in hippocampal pyramidal neuron cultures: Protective action of TGF-beta1. The Journal of Neuroscience 16: 4080–4088. 8753870 10.1523/JNEUROSCI.16-13-04080.1996 PMC2689548

[bibr46-1759091416679073] Michaud J. Fajardo R. Charron G. Sauvageau A. Berrada F. Ramla D. Kessous-Elbaz A. (2001) Neuropathology of NFHgp160 transgenic mice expressing HIV-1 env protein in neurons. Journal of Neuropathology and Experimental Neurology 60: 574–587. 11398834 10.1093/jnen/60.6.574

[bibr47-1759091416679073] Milligan E. D. O'Connor K. A. Armstrong C. B. Hansen M. K. Martin D. Tracey K. J. Watkins L. R. (2001) Systemic administration of CNI-1493, a p38 mitogen-activated protein kinase inhibitor, blocks intrathecal human immunodeficiency virus-1 gp120-induced enhanced pain states in rats. The Journal of Pain 2: 326–333. 14622812 10.1054/jpai.2001.26174

[bibr48-1759091416679073] Mitsuhashi S. Shima H. Li Y. Tanuma N. Okamoto T. Kikuchi K. Ubukata M. (2008) Tautomycetin suppresses the TNFalpha/NF-kappaB pathway via inhibition of IKK activation. International Journal of Oncology 33: 1027–1035. 18949366

[bibr49-1759091416679073] Morfini G. Burns M. R. Stenoien D. Brady S. T. (2012) Axonal transport. In: Brady S. T. Siegel G. Albers R. W. Price D. (eds) Basic neurochemistry: Principles of molecular, cellular and medical neurobiology, 8th ed Boston, MA: Elsevier, pp. 146–164.

[bibr50-1759091416679073] Morfini G. Pigino G. Beffert U. Busciglio J. Brady S. T. (2002) Fast axonal transport misregulation and Alzheimer's disease. Neuromolecular Medicine 2: 89–99. 12428805 10.1385/NMM:2:2:089

[bibr51-1759091416679073] Morfini G. Pigino G. Opalach K. Serulle Y. Moreira J. E. Sugimori M. Brady S. T. (2007) 1-methyl-4-phenylpyridinium affects fast axonal transport by activation of caspase and protein kinase C. Proceedings of the National Academy of Sciences of the United States of America 104: 2442–2447. 17287338 10.1073/pnas.0611231104 PMC1892945

[bibr52-1759091416679073] Morfini G. Pigino G. Szebenyi G. You Y. Pollema S. Brady S. T. (2006) JNK mediates pathogenic effects of polyglutamine-expanded androgen receptor on fast axonal transport. Nature Neuroscience 9: 907–916. 16751763 10.1038/nn1717

[bibr53-1759091416679073] Morfini G. Szebenyi G. Brown H. Pant H. C. Pigino G. DeBoer S. Brady S. T. (2004) A novel CDK5-dependent pathway for regulating GSK3 activity and kinesin-driven motility in neurons. The EMBO Journal 23: 2235–2245. 15152189 10.1038/sj.emboj.7600237 PMC419914

[bibr54-1759091416679073] Morfini G. A. Bosco D. A. Brown H. Gatto R. Kaminska A. Song Y. Brady S. T. (2013) Inhibition of fast axonal transport by pathogenic SOD1 involves activation of p38 MAP kinase. PLoS One 8: e65235. 23776455 10.1371/journal.pone.0065235 PMC3680447

[bibr55-1759091416679073] Morfini G. A. Burns M. Binder L. I. Kanaan N. M. LaPointe N. Bosco D. A. Brady S. T. (2009) Axonal transport defects in neurodegenerative diseases. The Journal of Neuroscience 29: 12776–12786. 19828789 10.1523/JNEUROSCI.3463-09.2009 PMC2801051

[bibr56-1759091416679073] Morfini G. A. You Y. M. Pollema S. L. Kaminska A. Liu K. Yoshioka K. Brady S. T. (2009) Pathogenic huntingtin inhibits fast axonal transport by activating JNK3 and phosphorylating kinesin. Nature Neuroscience 12: 864–871. 19525941 10.1038/nn.2346 PMC2739046

[bibr57-1759091416679073] Morris J. R. Lasek R. J. (1984) Monomer-polymer equilibria in the axon: Direct measurement of tubulin and actin as polymer and monomer in axoplasm. The Journal of Cell Biology 98: 2064–2076. 6202702 10.1083/jcb.98.6.2064 PMC2113063

[bibr58-1759091416679073] Munch C. Rosenbohm A. Sperfeld A. D. Uttner I. Reske S. Krause B. J. Ludolph A. C. (2005) Heterozygous R1101K mutation of the DCTN1 gene in a family with ALS and FTD. Annals of Neurology 58: 777–780. 16240349 10.1002/ana.20631

[bibr59-1759091416679073] Munoz L. Ralay Ranaivo H. Roy S. M. Hu W. Craft J. M. McNamara L. K. Watterson D. M. (2007) A novel p38 alpha MAPK inhibitor suppresses brain proinflammatory cytokine up-regulation and attenuates synaptic dysfunction and behavioral deficits in an Alzheimer's disease mouse model. Journal of Neuroinflammation 4: 21. 17784957 10.1186/1742-2094-4-21 PMC2014744

[bibr60-1759091416679073] Nath A. (2002) Human immunodeficiency virus (HIV) proteins in neuropathogenesis of HIV dementia. The Journal of Infectious Diseases 186(Suppl 2): S193–S198. 12424697 10.1086/344528

[bibr61-1759091416679073] Oh S. K. Cruikshank W. W. Raina J. Blanchard G. C. Adler W. H. Walker J. Kornfeld H. (1992) Identification of HIV-1 envelope glycoprotein in the serum of AIDS and ARC patients. Journal of Acquired Immune Deficiency Syndromes 5: 251–256. 1740750

[bibr62-1759091416679073] Oh S. B. Tran P. B. Gillard S. E. Hurley R. W. Hammond D. L. Miller R. J. (2001) Chemokines and glycoprotein120 produce pain hypersensitivity by directly exciting primary nociceptive neurons. The Journal of Neuroscience 21: 5027–5035. 11438578 10.1523/JNEUROSCI.21-14-05027.2001 PMC6762869

[bibr63-1759091416679073] Phillips T. J. Cherry C. L. Cox S. Marshall S. J. Rice A. S. (2010) Pharmacological treatment of painful HIV-associated sensory neuropathy: A systematic review and meta-analysis of randomised controlled trials. PLoS One 5: e14433. 21203440 10.1371/journal.pone.0014433 PMC3010990

[bibr64-1759091416679073] Pigino G. Morfini G. Atagi Y. Deshpande A. Yu C. Jungbauer L. Brady S. (2009) Disruption of fast axonal transport is a pathogenic mechanism for intraneuronal amyloid beta. Proceedings of the National Academy of Sciences of the United States of America 106: 5907–5912. 19321417 10.1073/pnas.0901229106 PMC2667037

[bibr65-1759091416679073] Platholi J. Federman A. Detert J. A. Heerdt P. Hemmings H. C. Jr. (2014) Regulation of protein phosphatase 1I by Cdc25C-associated kinase 1 (C-TAK1) and PFTAIRE protein kinase. Journal of Neurochemistry 289: 23893–23900. 10.1074/jbc.M114.557744 PMC4156073 25028520

[bibr66-1759091416679073] Politi C. Del Turco D. Sie J. M. Golinski P. A. Tegeder I. Deller T. Schultz C. (2008) Accumulation of phosphorylated I kappaB alpha and activated IKK in nodes of Ranvier. Neuropathology and Applied Neurobiology 34: 357–365. 17986183 10.1111/j.1365-2990.2007.00901.x

[bibr67-1759091416679073] Puls I. Jonnakuty C. LaMonte B. H. Holzbaur E. L. Tokito M. Mann E. Fischbeck K. H. (2003) Mutant dynactin in motor neuron disease. Nature Genetics 33: 455–456. 12627231 10.1038/ng1123

[bibr68-1759091416679073] Reid E. Kloos M. Ashley-Koch A. Hughes L. Bevan S. Svenson I. K. Marchuk D. A. (2002) A kinesin heavy chain (KIF5A) mutation in hereditary spastic paraplegia (SPG10). American Journal of Human Genetics 71: 1189–1194. 12355402 10.1086/344210 PMC385095

[bibr69-1759091416679073] Robinson B. Li Z. Nath A. (2007) Nucleoside reverse transcriptase inhibitors and human immunodeficiency virus proteins cause axonal injury in human dorsal root ganglia cultures. Journal of Neurovirology 13: 160–167. 17505984 10.1080/13550280701200102

[bibr70-1759091416679073] Sagot Y. Vejsada R. Kato A. C. (1997) Clinical and molecular aspects of motoneurone diseases: Animal models, neurotrophic factors and Bcl-2 oncoprotein. Trends in Pharmacological Sciences 18: 330–337. 9345852 10.1016/s0165-6147(97)01094-8

[bibr71-1759091416679073] Sakurai H. (2012) Targeting of TAK1 in inflammatory disorders and cancer. Trends in Pharmacological Sciences 33: 522–530. 22795313 10.1016/j.tips.2012.06.007

[bibr72-1759091416679073] Schneider J. Kaaden O. Copeland T. D. Oroszlan S. Hunsmann G. (1986) Shedding and interspecies type sero-reactivity of the envelope glycopolypeptide gp120 of the human immunodeficiency virus. The Journal of General Virology 67(Pt 11): 2533–2538. 2431105 10.1099/0022-1317-67-11-2533

[bibr73-1759091416679073] Schultz C. Konig H. G. Del Turco D. Politi C. Eckert G. P. Ghebremedhin E. Deller T. (2006) Coincident enrichment of phosphorylated IkappaBalpha, activated IKK, and phosphorylated p65 in the axon initial segment of neurons. Molecular and Cellular Neurosciences 33: 68–80. 16875840 10.1016/j.mcn.2006.06.008

[bibr74-1759091416679073] Shastry P. Basu A. Rajadhyaksha M. S. (2001) Neuroblastoma cell lines—A versatile in vitro model in neurobiology. The International Journal of Neuroscience 108: 109–126. 11328706 10.3109/00207450108986509

[bibr75-1759091416679073] Singh I. N. El-Hage N. Campbell M. E. Lutz S. E. Knapp P. E. Nath A. Hauser K. F. (2005) Differential involvement of p38 and JNK MAP kinases in HIV-1 Tat and gp120-induced apoptosis and neurite degeneration in striatal neurons. Neuroscience 135: 781–790. 16111829 10.1016/j.neuroscience.2005.05.028 PMC4310730

[bibr76-1759091416679073] Singh I. N. Goody R. J. Dean C. Ahmad N. M. Lutz S. E. Knapp P. E. Hauser K. F. (2004) Apoptotic death of striatal neurons induced by human immunodeficiency virus-1 Tat and gp120: Differential involvement of caspase-3 and endonuclease G. Journal of Neurovirology 10: 141–151. 15204919 10.1080/13550280490441103 PMC4309288

[bibr77-1759091416679073] Song Y. Brady S. T. (2013) Analysis of microtubules in isolated axoplasm from the squid giant axon. Methods in Cell Biology 115: 125–137. 23973070 10.1016/B978-0-12-407757-7.00009-8 PMC4460999

[bibr78-1759091416679073] Song Y. Kang M. Morfini G. Brady S. T. (2016) Fast axonal transport in isolated axoplasm from the squid giant axon. Methods in Cell Biology 131: 331–348. 26794522 10.1016/bs.mcb.2015.07.004

[bibr79-1759091416679073] Szebenyi G. Morfini G. A. Babcock A. Gould M. Selkoe K. Stenoien D. L. Brady S. T. (2003) Neuropathogenic forms of huntingtin and androgen receptor inhibit fast axonal transport. Neuron 40: 41–52. 14527432 10.1016/s0896-6273(03)00569-5

[bibr80-1759091416679073] Takekawa M. Tatebayashi K. Saito H. (2005) Conserved docking site is essential for activation of mammalian MAP kinase kinases by specific MAP kinase kinase kinases. Molecular Cell 18: 295–306. 15866172 10.1016/j.molcel.2005.04.001

[bibr81-1759091416679073] Toggas S. M. Masliah E. Rockenstein E. M. Rall G. F. Abraham C. R. Mucke L. (1994) Central nervous system damage produced by expression of the HIV-1 coat protein gp120 in transgenic mice. Nature 367: 188–193. 8114918 10.1038/367188a0

[bibr82-1759091416679073] Wilkerson J. L. Gentry K. R. Dengler E. C. Wallace J. A. Kerwin A. A. Armijo L. M. Milligan E. D. (2012) Intrathecal cannabilactone CB(2)R agonist, AM1710, controls pathological pain and restores basal cytokine levels. Pain 153: 1091–1106. 22425445 10.1016/j.pain.2012.02.015 PMC3603341

[bibr83-1759091416679073] Yi Y. Lee C. Liu Q. H. Freedman B. D. Collman R. G. (2004) Chemokine receptor utilization and macrophage signaling by human immunodeficiency virus type 1 gp120: Implications for neuropathogenesis. Journal of Neurovirology 10(Suppl 1): 91–96. 14982745 10.1080/753312758

[bibr84-1759091416679073] Zheng J. Ghorpade A. Niemann D. Cotter R. L. Thylin M. R. Epstein L. Gendelman H. E. (1999) Lymphotropic virions affect chemokine receptor-mediated neural signaling and apoptosis: Implications for human immunodeficiency virus type 1-associated dementia. Journal of Virology 73: 8256–8267. 10482576 10.1128/jvi.73.10.8256-8267.1999 PMC112843

[bibr85-1759091416679073] Zheng W. Ouyang H. Zheng X. Liu S. Mata M. Fink D. J. Hao S. (2011) Glial TNFalpha in the spinal cord regulates neuropathic pain induced by HIV gp120 application in rats. Molecular Pain 7: 40. 21599974 10.1186/1744-8069-7-40 PMC3121595

